# Relationship Between Endothelial and Angiogenesis Biomarkers Envisage Mortality in a Prospective Cohort of COVID-19 Patients Requiring Respiratory Support

**DOI:** 10.3389/fmed.2022.826218

**Published:** 2022-03-16

**Authors:** Felipe Maldonado, Diego Morales, Catalina Díaz-Papapietro, Catalina Valdés, Christian Fernandez, Nicolas Valls, Marioli Lazo, Carolina Espinoza, Roberto González, Rodrigo Gutiérrez, Álvaro Jara, Carlos Romero, Oscar Cerda, Mónica Cáceres

**Affiliations:** ^1^Department of Anaesthesia and Perioperative Medicine, Faculty of Medicine, Hospital Clínico de la Universidad de Chile, Universidad de Chile, Santiago, Chile; ^2^Program of Cellular and Molecular Biology, Institute of Biomedical Sciences (ICBM), Faculty of Medicine, Universidad de Chile, Santiago, Chile; ^3^Critical Care Unit, Hospital Clínico Universidad de Chile, Santiago, Chile; ^4^Emergency Department, Hospital Clínico Universidad de Chile, Santiago, Chile; ^5^Centro de Investigación Clínica Avanzada, Faculty of Medicine, Hospital Clínico de la Universidad de Chile, Universidad de Chile, Santiago, Chile; ^6^Millennium Nucleus of Ion Channel-Associated Diseases, Santiago, Chile; ^7^Millennium Institute on Immunology and Immunotherapy, Santiago, Chile

**Keywords:** angiogenesis, syndecan-1, angiopoietin-2, VEGF, COVID-19

## Abstract

**Purpose:**

Endothelial damage and angiogenesis are fundamental elements of neovascularisation and fibrosis observed in patients with coronavirus disease 2019 (COVID-19). Here, we aimed to evaluate whether early endothelial and angiogenic biomarkers detection predicts mortality and major cardiovascular events in patients with COVID-19 requiring respiratory support.

**Methods:**

Changes in serum syndecan-1, thrombomodulin, and angiogenic factor concentrations were analysed during the first 24 h and 10 days after COVID-19 hospitalisation in patients with high-flow nasal oxygen or mechanical ventilation. Also, we performed an exploratory evaluation of the endothelial migration process induced by COVID-19 in the patients' serum using an endothelial cell culture model.

**Results:**

In 43 patients, mean syndecan-1 concentration was 40.96 ± 106.9 ng/mL with a 33.9% increase (49.96 ± 58.1 ng/mL) at day 10. Both increases were significant compared to healthy controls (Kruskal–Wallis *p* < 0.0001). We observed an increase in thrombomodulin, Angiopoietin-2, human vascular endothelial growth factor (VEGF), and human hepatocyte growth factor (HGF) concentrations during the first 24 h, with a decrease in human tissue inhibitor of metalloproteinases-2 (TIMP-2) that remained after 10 days. An increase in human Interleukin-8 (IL-8) on the 10th day accompanied by high HGF was also noted. The incidence of myocardial injury and pulmonary thromboembolism was 55.8 and 20%, respectively. The incidence of in-hospital deaths was 16.3%. Biomarkers showed differences in severity of COVID-19. Syndecan-1, human platelet-derived growth factor (PDGF), VEGF, and Ang-2 predicted mortality. A multiple logistic regression model with TIMP-2 and PDGF had positive and negative predictive powers of 80.9 and 70%, respectively, for mortality. None of the biomarkers predicted myocardial injury or pulmonary thromboembolism. A proteome profiler array found changes in concentration in a large number of biomarkers of angiogenesis and chemoattractants. Finally, the serum samples from COVID-19 patients increased cell migration compared to that from healthy individuals.

**Conclusion:**

We observed that early endothelial and angiogenic biomarkers predicted mortality in patients with COVID-19. Chemoattractants from patients with COVID-19 increase the migration of endothelial cells. Trials are needed for confirmation, as this poses a therapeutic target for SARS-CoV-2.

## Introduction

Aggressive and rapidly evolving symptoms characterise a subset of patients with coronavirus disease 2019 (COVID-19). Early recognition of evolution is still not possible (1–3). Long-lasting hospitalisation due to prolonged mechanical ventilation is associated with altered oxygen diffusion in lung capillaries, which may be partly due to an increase in fibrotic areas (4). Angiopoietins are critical players in vessel maturation and mediate the migration, adhesion, and survival of endothelial cells. In conjunction with vascular endothelial growth factor (VEGF), angiopoietins promote neovascularisation (5). Furthermore, neovascularisation and fibrosis are present in the lungs of patients with COVID-19 (6, 7). Endothelial damage and angiogenesis are fundamental elements of this process (8–11), where intussusceptive and sprouting angiogenesis observed in autopsies reflect rapid vascular activation and proliferation during the disease (7, 12).

Elevation of the endothelial injury biomarkers, syndecan-1 and thrombomodulin (TM) in patients with sepsis is associated with intensive care unit (ICU) mortality (13, 14). In COVID-19, reports of syndecan-1 increase are related to disease severity and have been suggested as an assessment of the clinical course of the patient (15, 16). Soluble TM plasma elevation is also related to increased mortality in patients with COVID-19, as it marks direct endothelial cell damage (17, 18).

Besides injury, endothelial angiogenic activation poses another pathophysiological feature as well as a therapeutic opportunity. It seems to be a rapid phenomenon upon severe acute respiratory syndrome coronavirus-2 (SARS-CoV-2) infection, as this feature is observed in lung autopsies from non-surviving patients within 10 days of hospitalisation (7). Increased angiogenic biomarkers are associated with ICU admission and patients' reduced respiratory system compliance (19). Molecules such as VEGF and angiopoietin-2 (Ang-2) are associated with angiogenesis. Hepatocyte growth factor (HGF) stimulates cell migration and branching and acts synergistically with VEGF to promote new blood vessel formation, pericyte migration, and endothelial cell migration. Tissue inhibitor of metalloproteinases-2 (TIMP-2) binds to metalloproteinases (MMPs) and decreases extracellular matrix degradation (20).

Considering the brief instauration of the endothelial and angiogenic processes, we aimed to evaluate whether there is an early increase in endothelial and angiogenic biomarkers and whether early detection of these biomarkers is associated with mortality and major cardiovascular events in patients with COVID-19.

## Materials and Methods

We performed a single-centre prospective cohort study of patients with COVID-19 hospitalised at the Hospital Clínico de la Universidad de Chile. The study was approved by the ethics committee of our centre (Ref: OAIC 1161/20), registered online at https://www.clinicaltrials.gov (Ref: NCT04609332), and conducted according to the principles of the Helsinki Declaration under monitoring by the Good Clinical Practice unit of our institution. Written informed consent was obtained from all patients who were able to sign or from a legal representative if they were unable to provide consent. We adhered to the STROBE guidelines for reporting observational studies (21). The study was performed before vaccination campaigns in our country.

We defined changes in syndecan-1 blood concentrations during hospitalisation as the primary outcome. First, serum syndecan-1 concentrations were analysed during the first day of hospitalisation. Second, in the blood samples, we determined the concentrations of TM and a set of angiogenic factors as markers of profound endothelial damage and activation. After 10 days, if the patient remained hospitalised, a second biomarker set measurement was performed in this subgroup of patients. After 6 months of follow-up, mortality and major cardiovascular events were recorded. Finally, using an endothelial cell culture model, we performed an exploratory evaluation of the endothelial migration process in the serum samples of patients with COVID-19.

### Participants

We included patients aged 18 years and older with clinically suspected and laboratory reverse transcriptase-polymerase chain reaction (RT-PCR)-confirmed SARS-CoV-2 infection, hospitalised in critical patient care units with the need for high-flow nasal oxygen (HFNO) or mechanical ventilation during the first 24 h after arriving at our centre. The exclusion criteria were symptomatic patients with a negative RT-PCR for SARS-CoV-2 and patients who were treated with anticoagulants for a pre-existing comorbidity. Recruitment was performed between December 2020 and March 2021. For serum endothelial damage and angiogenic biomarkers, 10 mL blood samples were collected during the first 24 h and on the 10th day of hospitalisation. Hospitalisation clinical data were collected, and a telephonic follow-up was performed until the 6th month after hospital admission. As for control group, we included nine blood samples from healthy volunteers who did not present COVID-19 disease. They were recruited from the hospital and research laboratory during the study period. All patients' samples were processed similarly.

### Variables

For the primary outcome, increased and differences in syndecan-1 concentrations were analysed during the first 24 h and at the 10th day of hospitalisation. Blood serum concentrations of TM, human Ang-2, human HGF, human Interleukin-8 (IL-8), human platelet-derived growth factor (PDGF), human TIMP-2, and human VEGF were measured. Demographic, clinical, and laboratory data were obtained from the patients' medical charts. Major cardiovascular events were defined as death, the presence of pulmonary thromboembolism, and myocardial injury in patients with high-sensitive cardiac troponin I (Hs-cTn) elevation above 11 ng/L [99th percentile upper reference limit (URL)] (Vitros^®^, Ortho Clinical Diagnostics, UK) (22). Deaths outside the hospital were obtained from the National Registry of Deaths accessed online (https://www.registrocivil.cl). Surviving patients were contacted within the next 6 months after hospitalisation for clinical follow-up. As a definition of COVID-19, we analysed our cohort according to the National Institutes of Health clinical spectrum (23). We defined severity according to the need for ventilatory support and hospitalisation period as follows: severe, patients requiring HFNO ventilation who did not progress into shock or respiratory failure during the first 10 days of hospitalisation; critical, patients with the need for HFNO or mechanical ventilation due to respiratory failure, shock, or multiorgan dysfunction; and deceased patients.

Laboratory processing and outcome assessors were blinded to the patients. At study termination, the outcome assessor attained the patients' sample codes and performed the final analysis. After blinded assessments, we performed an exploratory analysis of serum from healthy patients and patients with severe COVID-19. Serum samples were used for *in vitro* endothelial migration assays.

### Sample Size

By the time of the study design, we found no previous data on syndecan-1 in patients with COVID-19; therefore, we selected a sample size of 40 patients for convenience of the primary outcome. Nevertheless, considering a normal syndecan-1 value of 31.6 ng/mL, a standard deviation of 15.3 ng/mL, our sample size calculation allowed us to detect a 29.7% change in the mean concentration of syndecan-1, with an alpha of 0.05 and a power of 80% (24, 25). Considering a 10% loss of patients, 44 patients were required for the two-sided test analysis.

### Statistical Methods

Categorical variables were summarised as relative frequencies. Continuous variables for primary and secondary outcomes were expressed as the mean and standard deviation (SD) or median and interquartile rank (IQR). Non-paired results were compared using the Mann–Whitney test. For group comparisons, we used the Kruskal–Wallis test with Dunn's multiple comparison test. The Wilcoxon test was used for paired data. Receiver operating characteristic (ROC) curves were generated for all biomarkers. The area under the curve (AUC) was calculated using the Youden index for cut-off values (26, 27). Multiple logistic regression was used with survivors and non-survivors as dichotomised variables as outcomes. We obtained a pseudo R2 (Tjur's R2) and used a Hosmer–Lemeshow goodness-of-fit and log-likelihood ratio test for hypothesis testing. Two-tailed *P*-values < 0.05, were considered significant for all analyses. Data were analysed using GraphPad Prism software (version 9.0; La Jolla, CA, USA).

### Quantification of Biomarkers

All blood samples were collected in a 15 mL centrifugation tube without heparin by an anaesthesiologist and coded before delivering the samples to the processing laboratory. All blood samples were immediately incubated for 1 h at 37°C and centrifuged at 400 g for 10 min. Blood serum was stored in a −80°C freezer for the final analysis (28).

To characterise the endothelial damage, we measured one glycocalyx biomarker (syndecan-1) and one endothelial membrane biomarker (TM). For biomarker levels in blood serum, we used the following enzyme-linked immunosorbent assay (ELISA): DuoSet Human Syndecan-1 (#DY2780; R&D Technologies, MN, USA) using blood samples diluted 20 times and five times, and human TM/BDCA−3 (#DTHBDO; R&D Technologies, MN, USA) using blood samples diluted six times. All measurements were performed in duplicate in one assay. Assays and analyses were performed according to the manufacturer's instructions (25).

To quantify Ang-2, HGF, IL-8, PDGF, TIMP-2, and VEGF, we used Q-PlexTM Human Angiogenesis (#150233HU; Quansys, Cellus, Santiago, Chile). The blood samples were diluted six times. Assays and analyses were performed according to the manufacturer's instructions. Additionally, a Proteome Profiler Human Angiogenesis Array kit (#ARY007, R&D Technologies) was used to identify 55 different angiogenesis proteins in COVID-19 and in the healthy volunteers' blood serum.

### Cell Migration

Cell migration was assayed using Transwell chambers (#3422; Costar, Corning Incorporated, ME, USA) with 8.0 μm-pore polycarbonate filters. 10,000 EA. hy926 cells were suspended in serum-free Dulbecco's Modified Eagle Medium (DMEM) without antibiotics and seeded in the upper compartment of the chamber. Ten samples of 10% v/v blood (five in the first 24 h and five on the 10th day of hospitalisation) from five randomly selected patients with COVID-19 and five from healthy individuals were added to the lower compartment of the chamber. Migration was allowed to occur for 24 h. Following the removal of the non-invading cells, the invading cells were fixed and stained with 0.2% crystal violet. Cell migration was evaluated by counting five (× 20) fields per chamber (29).

## Results

Forty-three patients were included between December 2020 and March 2021 (Supplementary Figure 1). The median age was 62 years (IQR 53–72); 39.5% were female and 60.5% were male. The mean body mass index was 26.4 kg/m^2^. The most prevalent comorbidities were hypertension (51%), diabetes (37%), obesity (12%), and dyslipidaemia (12%) (Table 1; Supplementary Table 1). For major cardiovascular events, we found a 44.2% (*n* = 19) incidence of myocardial injury in patients presenting to the hospital (Hs-cTn above URL), which increased to 55.8% (*n* = 24) of patients during hospitalisation. The incidence of pulmonary thromboembolism (PTE) was 9.3% (*n* = 4) in the first computed tomography angiography and increased during hospitalisation to 20% (*n* = 9) (Table 1). The incidence of in-hospital death was 16.3%. During the 6-month follow-up period, we found no out-of-hospital mortality.

**Table 1:** Patients characteristics.

		**COVID-19 severity**			
**Baseline values**	**Total**	**Severe**	**Critical**	**Non-survivors**	***p*-value**
**Age—years**	
Median (IQR)	62 (53–72)	55 (38–68)	62 (55–74)	73 (60–74)	<0.0001^#^
Range	55	42	49	17	
**Sex—*****n*****/ total** ***n*** **(%)**	
Female	18/43 (42)	4/43 (9)	11/43 (26)	3/43 (7)	0.9110
Male	25/43 (58)	7/43 (16)	14/43 (33)	4/43 (9)	0.9110
**BMI—mean (SD)**	26.4 (8.6)	28.2 (3.7)	25.7 (10.6)	26.9 (2.9)	
**Comorbidities—*****n*****/ total** ***n*** **(%)**	
Obesity	5/43 (12)	3/43 (7)	1/43 (2)	1/43 (2)	0.0751
Coronary heart disease	4/43 (9)	2/43 (4)	1/43 (2)	1/43 (2)	0.3339
Heart failure	3/43 (7)	–	1/43 (2)	2/43 (4)	0.1801
Chronic kidney failure	3/43 (7)	–	3/43 (7)	–	0.3281
Acute kidney failure	–	–	–	–	–
Stroke	–	–	–	–	–
Vascular disease	–	–	–	–	–
COPD	3/43 (7)	1/43 (2)	1/43 (2)	1/43 (2)	0.5870
Liver disease	1/43 (2)	–	–	1/43 (7)	0.0669
Diabetes	16/43 (37)	4/43 (9)	9/43 (21)	3/43 (7)	0.9402
Hypertension	22/43 (51)	5/43 (12)	14/43 (32)	3/43 (7)	0.8619
Smoking	5/43 (12)	1/43 (2)	2/43 (4)	2/43 (4)	0.2920
Dyslipidemia	4/43 (9)	1/43 (2)	3/43 (7)	–	0.9434
**Laboratory—mean (SD)**	
D-Dimer (ng/mL)	1,558 (1,745)	1,090 (808)	1,844 (2,168)	1,240 (585)	0.4274
C-reactive protein (mg/L)	159 (100)	96 (69)	183 (109)	169 (61)	0.0341^*^
ProBNP (pg/mL)	749 (914)	256 (252)	875 (1,092)	939 (943)	0.0827
Procalcitonin (ng/mL)	0.27 (0.39)	0.09 (–)	0.45 (0.5)	0.09 (0.05)	0.7000
**Myocardial injury—*****n*** **(within subgroup %)**	
24 h	19 (44.2)	2 (18)	12 (46.1)	5 (71)	0.1371
Total hospitalisation	24 (55.8)	3 (27)	15 (58)	6 (85)	0.0418^&^
**High-sensitive cardiac troponin I—mean (SD)**	
24 h (mg/ml)	35.7 (89.8)	6.7 (6.6)	41.7 (108.9)	52.2 (72)	0.0348^*^ 0.0318^#^
Maximum (mg/ml)	114.5 (341.1)	12.4 (20.9)	149.6 (439.8)	149.5 (119.5)	0.0362^*^ 0.0032^#^
**Thromboembolism—*****n*****/ total** ***n*** **(%)**	
First 24 h	4/43 (9)	0/11 (0)	3/25 (7)	1/7 (14)	0.4684
During hospitalisation	9/43 (20)	1/11 (1)	5/25 (20)	3/7 (43)	0.2489
**Arterial blood gases—mean (SD)**
pH	7.43 (0.04)	7.43 (0.04)	7.43 (0.05)	7.42 (0.04)	0.6802
pCO_2_	32.1 (4.3)	32.8 (4.4)	31.9 (4.6)	31.5 (2.9)	0.8469
pO_2_	82.6 (28.19)	96.63 (35.9)	79.42 (23.0)	72.63 (28.5)	0.2816
BE	−2.39 (3.25)	−1.98 (3.07)	−2.30 (3.40)	−3.33 (3.24)	0.6541
HCO_3_	21.36 (2.99)	21.62 (3.17)	21.50 (3.04)	20.44 (2.79)	0.6171
FiO_2_	49.5 (2.99)	34.2 (12.80)	55.2 (29.08)	53.7 (29.40)	0.1385
PaFi	221.0 (132.7)	316.4(151.5)	184.6 (102.7)	190.7 (133.2)	0.0265^*^
**Use of vasopressors—*****n*****/ total** ***n*** **(%)**
First 24 h	4/43 (9)	0/11 (0)	2/25 (8)	2/7 (29)	0.1189
Between 24 h and 10th day	16/43 (44)	0/11 (0)	11/25 (44)	5/7 (71)	0.0052^*^^#^
At 10th day	7/43 (16)	0/11 (0)	6/25 (24)	0/7 (0)	0.0812

* *Severe vs. critical patients Kruskal-Wallis*.

& *Chi-square*.

# *Severe vs. non-survivors Kruskal-Wallis*.

**Figure 1:**
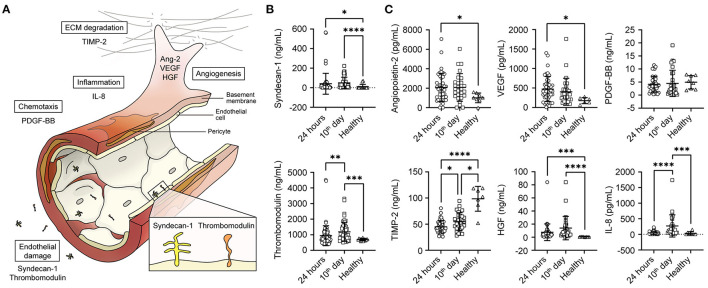
Serum levels of endothelial damage and angiogenesis-related proteins. **(A)** Summary of key molecules involved in angiogenesis and endothelial damage. **(B)** Graph representing the mean serum of syndecan-1 and thrombomodulin during the first 24 h of hospitalisation (*n* = 43), after 10 days (*n* = 28) and in healthy individuals (*n* = 9). **(C)** Graph representing the mean serum of angiopoietin-2, TIMP-2. VEGF, PDGF-BB, HGF, and IL-8. Statistical significance (*P*-values) is obtained using two-sided Kruskal–Wallis with Dunn's multiple comparison test, *P < 0.05; **P < 0.01; ***P < 0.001. ****P < 0.0001.

Key molecules are involved in endothelial and angiogenic processes (Figure 1A). The endothelial damage in patients with COVID-19 was characterised by a mean syndecan-1 concentration during the first 24 h of hospitalisation (40.96 ± 106.9 ng/mL) and, in the next 10 days, the subgroup of patients that remain hospitalised presented a 33.9% increase in serum concentrations (49.96 ± 58.1 ng/mL). Both increases were significant compared to healthy controls (Kruskal–Wallis *p* < 0.0001) (Intra-assay Coefficient of Variation of 6.9%). The mean TM level significantly increased from 942 ± 638 ng/mL in the first 24 h to 1,189 ± 608 ng/mL on the 10th day of hospitalisation and was different from that in healthy controls (Kruskal–Wallis *p* < 0.0001) (Figure 1B).

The angiogenic biomarker profile was characterised by an increase in Ang-2, VEGF, and HGF concentrations during the first 24 h after arriving at the centre. We found a decrease in the MMP inhibitor MMP TIMP-2 concentrations, which remained in the subgroup of hospitalised patients after 10 days. In this subgroup of patients with COVID-19, we also observed higher IL-8 levels on the 10th day accompanied by high HGF values compared to those in healthy controls (Figure 1C).

**Figure 2:**
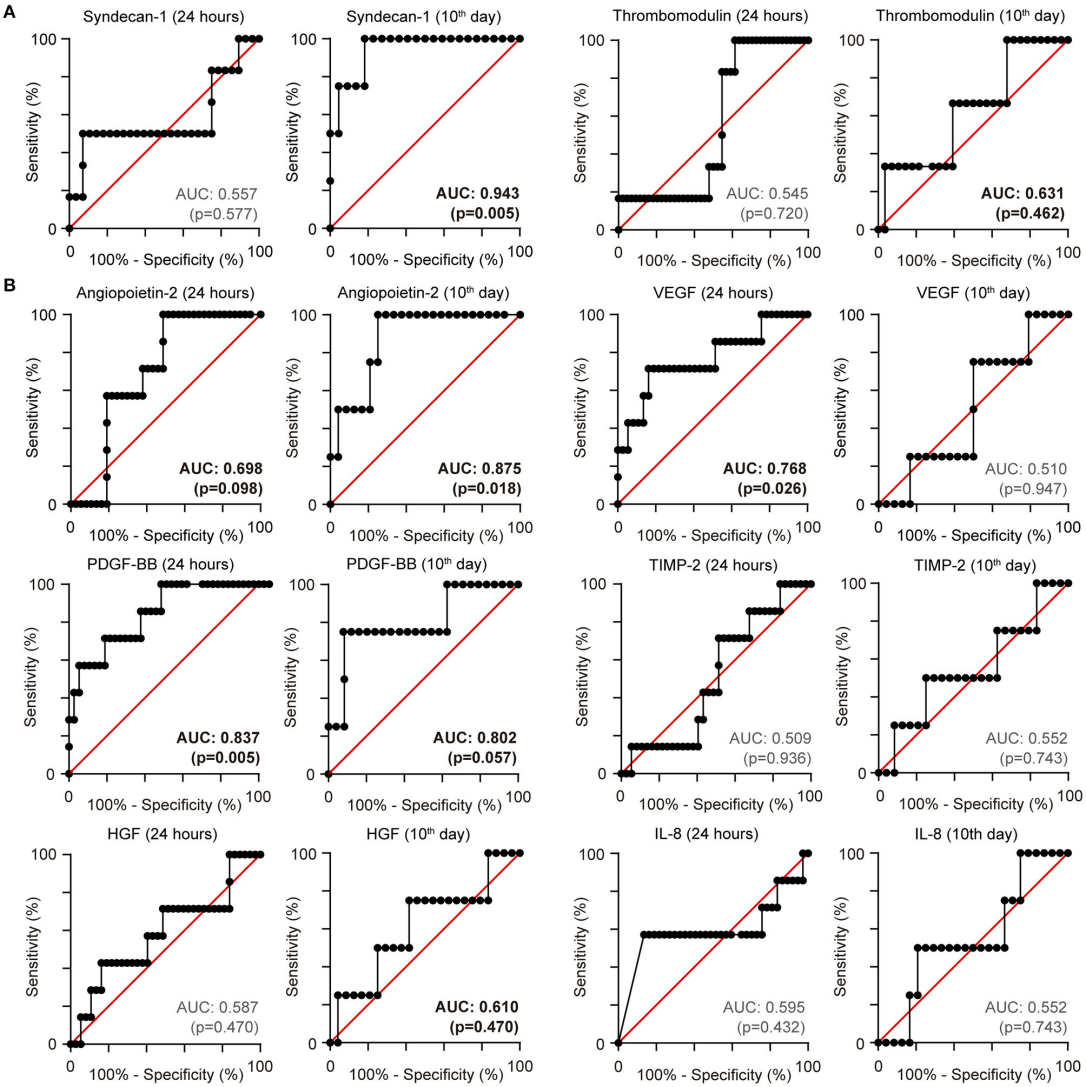
Receiver operating characteristic (ROC) curve for mortality: **(A)** Endothelial biomarkers **(B)** Angiogenic biomarkers. ROC curves with area under the curve for mortality.

For biomarkers obtained in the first 24 h, PDGF had an AUC of 0.838 (95% CI 0.69–0.99; *p* = 0.005) to predict mortality from survivors (cut-off value, 2,118 pg/mL; sensitivity, 71.43%; specificity, 81.08%). VEGF had an AUC of 0.768 (95% CI 0.56–0.98: *p* = 0.0257) to predict mortality (cut-off value, 266 pg/mL; sensitivity, 71.43%; specificity, 83.78%). On the 10th day of hospitalisation, Ang-2 had an AUC of 0.875 (95% CI 0.73–1.0; *p* = 0.018) to predict mortality from survivors (cut-off value, 2,388 pg/mL; sensitivity, 100%; specificity, 75%). Interestingly, syndecan-1 had an AUC of 0.94 (95% CI 0.84–1.0; *p* = 0.005) to predict mortality from survivors (cut-off value of 40.1 ng/mL; sensitivity of 100% and specificity of 81.82%) (Figures 2A,B). Finally, none of the biomarkers predicted myocardial injury or PTE.

**Figure 3:**
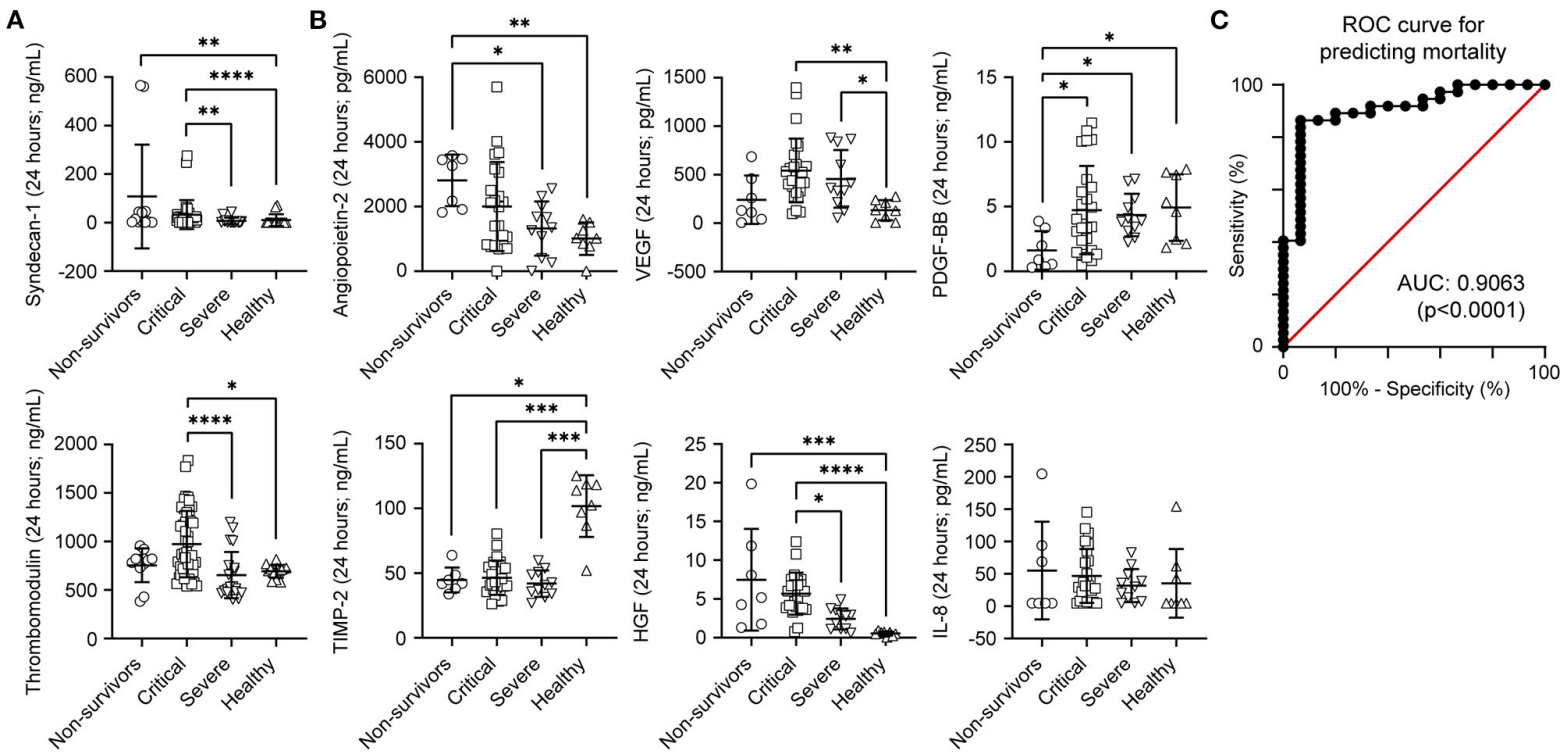
Serum levels comparison according to coronavirus disease 2019 severity during the first 24 h of hospitalisation. **(A)** Levels of syndecan-1 and thrombomodulin. **(B)** Graph representing the mean serum of angiopoietin-2, vascular endothelial growth factor (VEGF), human platelet-derived growth factor (PDGF), human tissue inhibitor of metalloproteinases-2 (TIMP-2), human hepatocyte growth factor (HGF), and human Interleukin-8 (IL-8). Statistical significance (*P*-values) is obtained using two-sided Kruskal–Wallis test, *P < 0.05; **P < 0.01; ***P < 0.001; ****P < 0.0001. **(C)** Multivariate model logistic regression receiver operating characteristic curve using TIMP-2 and PDGF for predicting mortality (*n* = 11 severe, *n* = 25 critical, *n* = 7 non-survivors).

According to severity definitions, we found that the concentrations of syndecan-1 in the first 24 h were significantly elevated in patients who developed a critical illness or died. TM levels were elevated in patients with severe and critical disease (Figure 3A). Patients who died from COVID-19 also presented with elevated Ang-2 and HGF levels, accompanied by low concentrations of TIMP-2. Critical disease was characterised by an increase in VEGF and HGF and a decrease in TIMP-2 (Figure 3B).

Next, we evaluated whether a combination of the biomarkers already shown may outperform the prediction accuracy for in-hospital mortality. We performed a multiple logistic regression model using Ang-2, HGF, IL-8, PDGF, TIMP-2, VEGF, syndecan-1, and TM values obtained within 24 h of the patient arriving at the hospital. A dual combination of VDGF, PDGF, and TIMP-2 improved our model. The combination of TIMP-2 and PDGF as predictors had a positive predictive power of 80.9% and a negative predictive power of 70% for mortality, with an AUC of 0.90 (95% CI 0.816–0.997; *p*-value < 0.0001; Tjur's R2 of 0.43), Hosmer–Lemeshow *p*-value of 0.34 and a log-likelihood ratio *p*-value < 0.0001 (Figure 3C).

**Figure 4:**
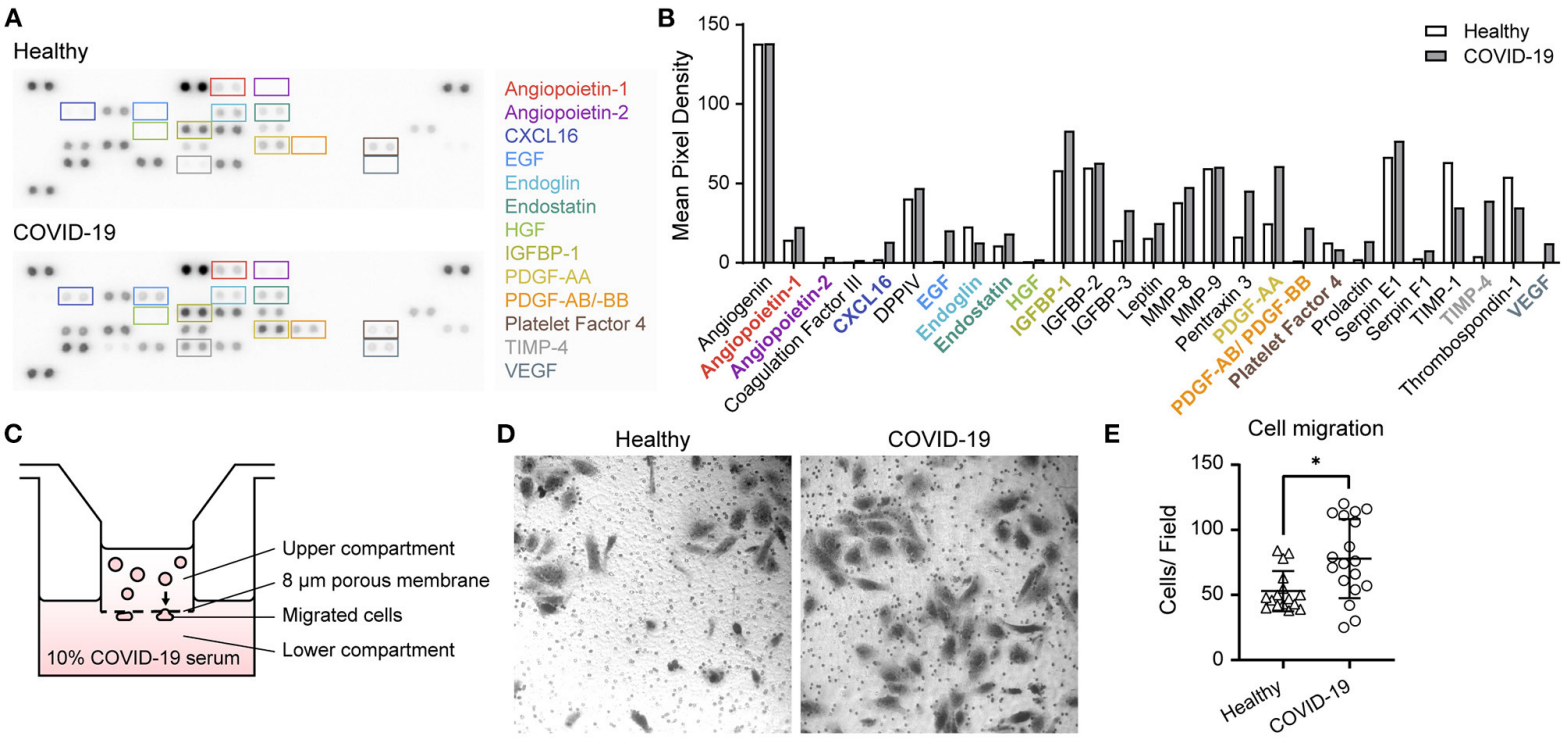
Proteome profiler array and cell migration induced by coronavirus disease 2019 blood serum. **(A)** Representative image of the angiogenic proteome profiler array using blood serum samples from healthy participants and from those with severe COVID-19. **(B)** Quantification of pixel mean density of each angiogenesis-related protein. **(C)** Schematics of Transwell Boyden chamber assay using blood serum samples from COVID-19-infected and healthy participants in the lower compartment as chemoattractants. **(D)** Representative images of migrated endothelial EA.hy926 cells induced by blood serum samples of COVID-19-infected and healthy participants. **(E)** Quantification of migrated cells by field (*n* = 5 healthy individuals, *n* = 3 severe, *n* = 1 critical, and *n* = 1 non-surviving COVID-19 patient) using both samples in first 24 h and on the 10th day of hospitalisation. Statistical significance (*P*-values) is obtained using Mann–Whitney *P < 0.05.

To further evaluate the biomarkers, we tested whether patients' serum samples were able to induce changes in endothelial function. To identify biomarkers that could be modulated by SARS-CoV-2, we used a semi-quantitative methodology, a proteome profiler array, using serum from patients with COVID-19 and healthy individuals. We found that Angiopoietin-1 (Ang-1), Ang-2, endostatin, VEGF, TIMP-1, TIMP-4, CXCL4, PDGF-AB/BB, TSP-1, EGF, CXCL16, and CD105 were modulated. Interestingly, Ang-1, Ang-2, VEGF, and CXCL-4 were involved in angiogenesis (Figures 4A,B) (30). Further, we evaluated whether blood serum from patients with COVID-19 and healthy individuals served as chemoattractants to endothelial EA.hy 926 cells by performing a Transwell assay (Figures 4C–E). We observed that blood serum from patients with COVID-19 showed increased cell migration compared to that in healthy blood serum.

## Discussion

COVID-19 is an aggressive disease. Here, we observed that endothelial injury and angiogenic biomarkers increased upon arrival of patients in need of high-flow nasal oxygen (HFNO) or mechanical ventilation support. An imbalance in the pro-angiogenic profile of endothelial activation is suggested in our migration and angiogenic assays using the serum samples of patients with COVID-19 as a chemotactic agent. Furthermore, an increase in biomarkers was predictive of mortality in our cohort.

The increase in syndecan-1 and TM levels confirmed substantial endothelial damage. As for angiogenesis, the sole elevation of one angiogenic protein has been described as insufficient for promoting endothelial cell survival and *in vitro* tubulogenesis, and the need for combination increases cell survival, tubulogenesis, and neovascularisation in rat corneas (20). In our cohort, we found an increase in VEGF, HGF in combination with high circulating Ang-2 levels. For PDGF, in deceased patients, we observed a decrease in the concentration in blood samples obtained upon arrival at the hospital. Also, we observed a decrease in TIMP-2 levels in the first 24 h and 10 days of hospitalisation, respectively. TIMP-2 inhibits VEGF-induced angiogenesis (31). Therefore, serum reduction reinforces the imbalance in the angiogenic profile of COVID-19 patients. Finally, IL-8 increase on the 10th day of hospitalisation adds a paracrine angiogenic factor that modulates the endothelial cell response (32, 33).

Using the proteome profiler array approximation, in patients with COVID-19 we confirmed the angiogenic and chemotactic serum profile compared to healthy volunteers of similar ages. Of the 55 molecules analysed, the observed increase in insulin-like growth factor binding proteins (IGFBPs), chemokines such as CXCL16 and Pentraxin-3, and endothelial growth factor (EGF) reaffirms the upregulation of angiogenic factors (34–39). There is compelling evidence that there is a need for crosstalk between different factors, as this has been described between HGF and VEGF, enhancing VEGF-driven angiogenesis; therefore, an increase in different molecules was expected (40). Finally, this profile was associated with *in vitro* endothelial cell migration, suggesting the ability of serum to activate endothelial functions.

SARS-CoV-2 infection through the angiotensin converting enzyme-2 (ACE-2) receptor makes COVID-19 systemic. Endothelial injury is a hallmark of tissue permeability, lung oedema, and organ dysfunction. Endothelial cells control vascular tone and permeability by inducing endothelium-derived relaxation and contractile factors. Upon activation, endothelial cells secrete chemoattractants, cytokines, and adhesion molecules (41). With dysfunction, endothelial cells fail to produce nitric oxide (NO), losing the suppression effect on activated molecule release, a feature observed in patients with COVID-19 (42, 43). Other endothelial functions, such as angiogenesis and cellular migration, have received less attention in the literature. When Ackerman et al. described intussusceptive and sprouting angiogenesis as a novel feature of SARS-CoV-2 infection, compared with autopsies from influenza patients, he showed a vessel proliferation that accompanied a rapid decline in lung function and death (7). This angiogenic progression has been primarily studied in tumours. Circulating VEGF, HGF, and Ang-2 levels have been described in breast cancer, hepatocarcinoma, and melanoma (44–46).

There is increasing evidence that endothelial damage is a predictor of outcomes. Smadja et al., in an observational cohort of 40 patients, found that Ang-2 concentration at admission is a relevant factor to predict transfer to ICU with an ROC of AUC 77.2 (80.1% sensitivity and 70% specificity) (19). Vassiliou et al., using endothelial biomarkers in hospitalised patients with COVID-19 admitted to the ICU, found that elevation of sE-selectin, sP-selectin, Ang-2, and sICAM-1 levels were significantly elevated in ICU non-survivors compared to survivors, with a higher mortality probability. In addition, sE-selectin, Ang-2, and sICAM-1 from the generated ROC curves were >0.85, indicating that elevated levels of these markers upon ICU admission could predict mortality in COVID-19 (47). Recently, de Moraes et al. described that angiopoietins, their receptors, and VEGF are associated with severity of COVID-19, suggesting that targeting the Ang/Tie2 and VEGF-A pathways could be valuable strategies to modulate COVID-19 severity (48). This reinforces our findings that endothelial damage is an early phenomenon, relates to hospital admission biomarker concentrations as a predictive tool for mortality, and can be a useful strategy for patient management.

Among the limitations of our study, we find the small number of participants and controls, which may reduce the strength of the statistical analysis. Although some of the biomarkers exhibited good AUC in the ROC curves and could predict disease mortality and severity in SARS-CoV-2, a larger trial is needed to confirm our results. Additionally, the number of samples obtained on day 10 was reduced due to deceased patients. Another limitation is the variation of absolute values between assays in the literature due to the temperature of sample management. Our samples were obtained with different processing methods than other laboratories, were we aimed to maintain normothermia to avoid platelet activation and angiogenic biomarkers release (49). Hence absolute values should be interpreted carefully and compared to studies with similar sample management. In relation to our migratory assay, we did not perform inhibitory experiments, and our findings need to be further investigated.

Finally, endothelial injury and angiogenesis biomarkers have been associated with mortality in patients with sepsis (14, 50). Here, we observed that early endothelial and angiogenic biomarkers increased the prediction of mortality, although they failed to predict myocardial injury and PET. Even though all patients in our cohort received the dexamethasone-recovery protocol (51), serum biomarkers remained altered on the 10th day of hospitalisation. The angiogenic profile associated with the known cytokine storm may be a relevant feature in COVID-19 induced organ dysfunction (52), and differences in sprouting or intussusceptive angiogenic evolution in different sepsis aetiologies may impact outcomes (7). Regarding the endothelial and angiogenic features of COVID-19, many questions still remain regarding their usefulness as biomarkers and the potential role of anti-angiogenic treatments in patients with the disease.

## Data Availability Statement

The original contributions presented in the study are included in the article/Supplementary Material, further inquiries can be directed to the corresponding author.

## Ethics Statement

The studies involving human participants were reviewed and approved by Ethics Committee of Hospital Clínico Universidad de Chile (Ref: OAIC 1161/20). The patients/participants provided their written informed consent to participate in this study. Written informed consent was obtained from all patients who were able to sign or from a legal representative when they were unable to provide consent.

## Author Contributions

FM and MC conceived the study, drafted the work, and performed the funding acquisition. FM, DM, CD-P, CV, CF, ML, CE, and MC performed the data collection. Data were analysed by FM, DM, CD-P, CF, NV, RGu, ÁJ, CR, OC, and MC and interpreted by FM, NV, RGo, RGu, ÁJ, CR, OC, and MC. All the authors revised the work, commented on previous versions of the manuscript, approved the version to be published, and agreed to be accountable for all aspects of the work in ensuring that questions related to the accuracy or integrity of any part of the work are appropriately investigated and resolved.

## Funding

This work was supported by the Priority Health Problems: COVID-19 Investigation Grant of the Hospital Clínico de la Universidad de Chile, Santiago, Chile (FM), and a grant from the National Fund for Science and Technology (FONDECYT) Grant 1181263 (MC) from the National Agency of Research and Development (ANID). The Millennium Nucleus of Ion Channel-Associated Diseases (MiNICAD) from the Millennium Initiative, ANID funds (OC and MC). FONDECYT 1200917 funds (OC).

## Conflict of Interest

The authors declare that the research was conducted in the absence of any commercial or financial relationships that could be construed as a potential conflict of interest.

## Publisher's Note

All claims expressed in this article are solely those of the authors and do not necessarily represent those of their affiliated organizations, or those of the publisher, the editors and the reviewers. Any product that may be evaluated in this article, or claim that may be made by its manufacturer, is not guaranteed or endorsed by the publisher.

## Supplementary Material

Supplementary Figure 1 Strobe diagram.

Supplementary Table 1 Healthy volunteers' characteristics.
