# Supplementary figures and images for: Relationship Between Endothelial and Angiogenesis Biomarkers Envisage Mortality in a Prospective Cohort of COVID-19 Patients Requiring Respiratory Support
Source: Front Med (Lausanne). 2022 Mar 16;9:826218. doi: 10.3389/fmed.2022.826218 (PMC8966493; doi:10.3389/fmed.2022.826218)

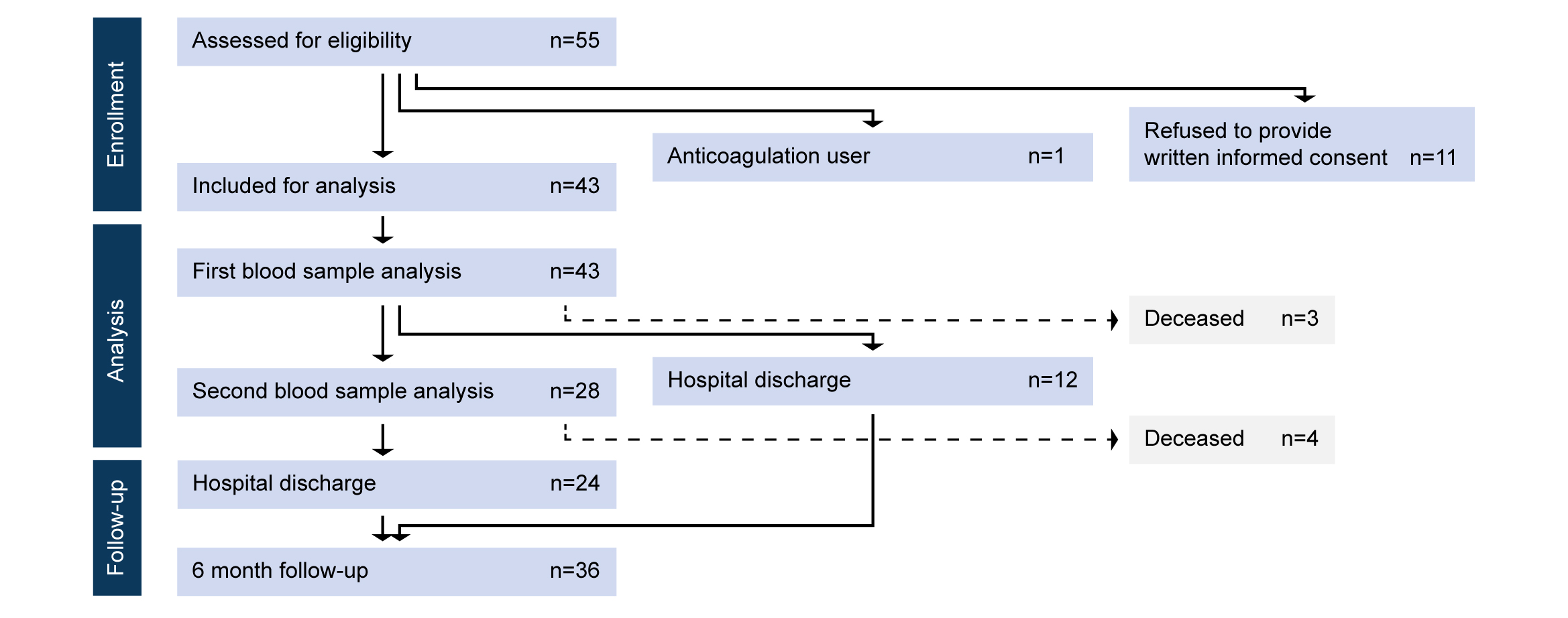

Supplement: Supplementary Figure 1 — Strobe diagram. [file Image_1.JPEG]
