# Supplementary material for: Relationship Between Endothelial and Angiogenesis Biomarkers Envisage Mortality in a Prospective Cohort of COVID-19 Patients Requiring Respiratory Support
Source: Front Med (Lausanne). 2022 Mar 16;9:826218. doi: 10.3389/fmed.2022.826218 (PMC8966493; doi:10.3389/fmed.2022.826218)
Supplement: Supplementary Table 1 — Healthy volunteers' characteristics. [file Table_1.pdf]

Table S1 Healthy volunteers' characteristics

**Age - Years**	
Median (IQR)	64 (35-66)
Range	40
**Sex - n/ total n (%)**	
Female	3/9 (33)
Male	6/9 (67)
**BMI - mean (SD)**	26.2 (4.34)
**Comorbidities - n/ total n (%)**	
Obesity	2/9 (22)
Coronary heart disease	0/9 (0)
Heart failure	0/9 (0)
Chronic kidney failure	0/9 (0)
Acute kidney failure	0/9 (0)
Stroke	0/9 (0)
Vascular disease	0/9 (0)
COPD	0/9 (0)
Liver disease	0/9 (0)
Diabetes	0/9 (0)
Hypertension	3/9 (33)
Smoking	0/9 (0)
Dyslipidemia	1/9 (11)
**Symptoms - n/ total n (%)**	0/9 (0)
